# Charleston Medical Journal and Review

**Published:** 1852-07

**Authors:** 


*The Charleston Medical Journal & Review,*—This is one of the largest Medical Periodicals in the United States, and has reached the third No. of volume seven. It has a large number of able contributors. The selections from foreign and domestic journals, is varied, and to meet the wants of the Profession. The journal is to us a regular visitor, and we are always glad to find it on our table. It is edited and published by D. J. Cain, M. D., and F. Peyre Percher, M. D., Charleston, South Carolina.

We would be glad to see every practitioner of Dental Surgery, not only supply himself with the Dental Publications, but also take some one of the many valuable Medical Periodicals of the land. In this way he might maintain a general knowledge of the prevailing diseases of the country, the history of the profession, &c., &c., all of which would be of vast service to him.

